# d-Allulose Inhibits Ghrelin-Responsive, Glucose-Sensitive and Neuropeptide Y Neurons in the Arcuate Nucleus and Central Injection Suppresses Appetite-Associated Food Intake in Mice

**DOI:** 10.3390/nu14153117

**Published:** 2022-07-29

**Authors:** Yermek Rakhat, Kentaro Kaneko, Lei Wang, Wanxin Han, Yutaka Seino, Daisuke Yabe, Toshihiko Yada

**Affiliations:** 1 Division of Integrative Physiology, Kansai Electric Power Medical Research Institute, Kyoto 604-8436, Japan; rakhatyermek@gmail.com (Y.R.); lei.wang@kepmri.org (L.W.); zhouxinxin981@gmail.com (W.H.); 2 Department of Diabetes, Endocrinology and Metabolism/Rheumatology and Clinical Immunology, Gifu University Graduate School of Medicine, Gifu 501-1194, Japan; daichan.yabechan@gmail.com; 3 Kobe Biotechnology Research and Human Resource Development Center, Kobe University Graduate School of Medicine, Kobe 650-0047, Japan; 4 Department of Agricultural Chemistry, School of Agriculture, Meiji University, Kanagawa 214-8571, Japan; kanekok@meiji.ac.jp; 5 Division of Food Science and Biotechnology, Graduate School of Agriculture, Kyoto University, Kyoto 611-0011, Japan; 6 Yutaka Seino Distinguished Center for Diabetes Research, Kansai Electric Power Medical Research Institute, Osaka 553-0003, Japan; yutaka.seino.hp@gmail.com

**Keywords:** d-allulose, ghrelin, glucose, arcuate nucleus, neuropeptide Y, cytosolic Ca^2+^, appetite, hunger, food intake, obesity, diabetes

## Abstract

d-allulose, a rare sugar, has sweetness with few calories. d-allulose regulates feeding and glycemia, and ameliorates hyperphagia, obesity and diabetes. All these functions involve the central nervous system. However, central mechanisms underlying these effects of d-allulose remain unknown. We recently reported that d-allulose activates the anorexigenic neurons in the hypothalamic arcuate nucleus (ARC), the neurons that respond to glucagon-like peptide-1 and that express proopiomelanocortin. However, its action on the orexigenic neurons remains unknown. This study investigated the effects of d-allulose on the ARC neurons implicated in hunger, by measuring cytosolic Ca^2+^ concentration ([Ca^2+^]_i_) in single neurons. d-allulose depressed the increases in [Ca^2+^]_i_ induced by ghrelin and by low glucose in ARC neurons and inhibited spontaneous oscillatory [Ca^2+^]_i_ increases in neuropeptide Y (NPY) neurons. d-allulose inhibited 10 of 35 (28%) ghrelin-responsive, 18 of 60 (30%) glucose-sensitive and 3 of 8 (37.5%) NPY neurons in ARC. Intracerebroventricular injection of d-allulose inhibited food intake at 20:00 and 22:00, the early dark phase when hunger is promoted. These results indicate that d-allulose suppresses hunger-associated feeding and inhibits hunger-promoting neurons in ARC. These central actions of d-allulose represent the potential of d-allulose to inhibit the hyperphagia with excessive appetite, thereby counteracting obesity and diabetes.

## 1. Introduction

The 1.9 billion (39%) of adults are obese or overweight and the numbers are still increasing in the world, producing an obesity pandemic [1]. Overeating and abnormal feeding rhythm lead to obesity [2]. d-allulose (d-psicose) is one of the rare sugars [3] and is present in limited amounts as a natural component of a few plants such as Itea plants (Zuina) [4] and particular bacteria [4]. d-allulose is a C-3 epimer of d-fructose and has sweetness equal to glucose and approximately 70% of fructose and sucrose, while having only 0.2 kcal/g compared to 4 kcal/g for glucose, fructose and sucrose [5].

d-allulose treatment promotes glucose tolerance in humans [6] and rodents including healthy mice [7], high fat diet (HFD)-induced obese (DIO) mice [7] and type-2 diabetic Otsuka Long-Evans Tokushima Fatty (OLETF) rats [8]. Regarding the underlying mechanisms, d-allulose inhibits the gut digestive enzymes and d-glucose absorption [9], and activates the glucokinase (GK) regulatory protein in the liver [10] in rodents. d-allulose reduces weight in OLETF rats [8], DIO mice [7,11] and in overweight subjects [12]. The weight-reducing effect partly contributes to the glycemic control by d-allulose [7]. Regarding the mechanisms underlying the weight reduction, d-allulose reduces enzyme activities involved in fatty acid synthesis and enhances energy expenditure in rats [13,14,15,16]. In addition, d-allulose modulates microbiota, diminishes inflammation and promotes the production of short-chain fatty acids (SCFA) in rodents [17]. We reported [7] that oral d-allulose decreases feeding and body weight and induces secretion of glucagon-like peptide-1 (GLP-1), an anorexigenic hormone, in lean and DIO mice, and that the secreted GLP-1 stimulates the vagal afferent nerves and induces the extracellular signal-regulated kinase (ERK) phosphorylation in the vagal afferent nodose ganglion and the brain stem, nucleus tractus solitarius [7]. This report also showed that a repeated once-daily d-allulose injection for 10 days reduced food intake and body weight in a long-lasting manner in DIO mice [7]. The d-allulose-induced GLP-1-vagal pathway is implicated in reductions of food intake and body weight. However, the effects of d-allulose on the central nervous system have long been unexplored.

We have recently shown that an intracerebroventricular (icv) injection of d-allulose suppressed food intake in mice [18]. Notably, d-allulose (5.6, 16.7 and 56 mM) concentration-dependently and osmolarity-independently activated anorexigenic neurons in the hypothalamic arcuate nucleus (ARC), GLP-1 responsive-neurons and proopiomelanocortin (POMC) neurons [18]. Furthermore, d-allulose cooperated with GLP-1 to activate ARC and POMC neurons [18]. The activation of these anorexigenic neurons could be implicated in the central action of d-allulose to inhibit feeding.

However, feeding behavior and weight are regulated by the balance of the anorexigenic neural system and the orexigenic neural system, both located in the ARC [19,20]. However, the effect of d-allulose on the ARC orexigenic neurons remains unknown. Accumulating evidence has demonstrated that the ARC neuron that co-expresses the neuropeptide Y (NPY) and agouti-related peptide (AgRP) (AgRP/NPY neuron) is necessary and sufficient to produce hunger. Fasting acutely activates the ARC NPY/AgRP neurons [21,22] and increases NPY content in paraventricular nucleus (PVN) [23]. Optogenetics-driven selective activation of NPY/AgRP neurons triggers feeding behavior [24,25], whereas selective deletion of NPY/AgRP neurons in adult mice rapidly reduces feeding and body weight, leading to death [26,27]. Ghrelin and lowered glucose concentration are the major factors that activate the orexigenic neurons in the ARC, including NPY/AgRP neurons [20,27,28].

This study explored the effect of d-allulose on the neurons that respond to ghrelin (ghrelin-responsive neurons), that respond to lowering glucose concentration (glucose-sensitive neurons), and that express NPY (NPY neurons) in the ARC, the neuron types that partly overlap with each other and serve as the principal neurons for producing hunger [20,28,29,30,31]. Cytosolic free Ca^2+^ concentration ([Ca^2+^]_i_) in single ARC neurons was measured by fura-2 microfluorometry. The effect of the central injection of d-allulose on feeding, with particular attention to that in the early dark phase when hunger is produced [2], was examined. We found that d-allulose depressed the ghrelin-induced and low glucose-induced [Ca^2+^]_i_ increases in ARC neurons and inhibited the spontaneous oscillatory increases in [Ca^2+^]_i_ in NPY neurons. Icv injection of d-allulose at 18.00 significantly reduced food intake at 20:00 and 22:00 in the early dark phase.

## 2. Materials and Methods

### 2.1. Chemicals

d-allulose (purity > 98%) was provided by Matsutani Chemical Industry Co., Ltd. (Hyogo, Japan). Ghrelin was obtained from Peptide Institute (Osaka, Japan). In [Ca^2+^]_i_ measurements; test agents were dissolved in HEPES-buffered Krebs-Ringer bicarbonate buffer (HKRB) solution composed of (in mM) 129 NaCl, 5.0 NaHCO_3_, 4.7 KCl, 1.2 KH_2_PO_4_, 1.8 CaCl_2_, 1.2 MgSO_4_ and 10 HEPES with pH adjusted at 7.4 using NaOH.

### 2.2. Animals

C57BL/6J mice were purchased from Japan SLC (Shizuoka, Japan), NPY-hrGFP mice (JAX stock number 006417) were kindly afforded by Dr. Joel Elmquist. Mice were housed under constant temperature (23 ± 1 °C) and humidity (55 ± 5%) with 12 h light/dark cycle (lights off at 19:00). Food and water were available ad libitum. For the study of [Ca^2+^]_i_ measurement, 4 to 5 mice were kept in a cage. For icv injection and feeding experiments, mice were kept in single cages. Animal experiments were performed following approval from the Institutional Animal Experiment Committee and in accordance with the Institutional Regulation for Animal Experiments at Kobe University (IACUC approval number; 30-10-06-R2).

### 2.3. Cannula Implantation and Icv Injection

Male mice were anesthetized with isoflurane and located in a stereotaxic frame. A 26-gauge single stainless-steel guide cannula (C315GS-5-SPC, Plastics One, Roanoke, VA, USA) was placed into the lateral ventricles (−0.45 mm from bregma, ±0.9 mm lateral and −2.5 mm from the skull). The cannula was fixed to the skull with screws and dental cement. The mice were housed in single cages, allowed to recover from the operation for 1 week, and then injected with saline (0.9% NaCl, 3 μL) or d-allulose (3 M, 3 μL) at 18.00.

### 2.4. Preparation of Single Neurons from ARC

The ARC of hypothalamus was isolated from the brain of 5–7 week-old male mice and single neurons were prepared following previous reports [31,32,33]. Briefly, mice were anesthetized with isoflurane and decapitated, and the brain was removed. Brain slices were prepared, and the ARC of left and right sides were punched out. The dissected tissues were incubated with 20 units/mL papain (Sigma Aldrich, St. Louis, MO, USA), 0.015 mg/mL deoxyribonuclease, and 0.75 mg/mL BSA in HKRB for 16 min at 36 °C, followed by gentle mechanical trituration for 5–10 min and centrifugation at 100× *g* for 5 min. The pellet was resuspended in HKRB and placed onto coverslips. The cells were kept at 30 °C under 100% moisture for 2–8 h until [Ca^2+^]_i_ measurements.

### 2.5. Measurements of [Ca^2+^]_i_ in Single ARC Neurons

[Ca^2+^]_i_ was measured by fura-2 microfluorometry, following previous reports [31,32,33]. Briefly, following incubation with 2 μM fura-2 AM (DOJINDO, Kumamoto, Japan) for 30 min at 30 °C, the cells were superfused at 30 °C at 1 mL/min with HKRB containing 2 mM glucose unless otherwise expressed. Test agents were given under superfusion conditions. Data were collected from the single cells that were identified as neurons according to reported criteria [31]; relatively large diameter (≥10 μm), clear and round cell bodies, and [Ca^2+^]_i_ responses to KCl. Fluorescence ratio (F340/F380) was produced by Aquacosmos ver. 2.5 (Hamamatsu Photonics, Shizuoka, Japan). The [Ca^2+^]_i_ increases with amplitudes twice larger than fluctuations of baseline or greater were considered as responses. The amplitude of [Ca^2+^]_i_ increases was calculated by subtracting the basal [Ca^2+^]_i_ level from the peak [Ca^2+^]_i_ level. In all experiments, neurons from at least three separate preparations were analyzed.

### 2.6. Statistical Analysis

All the data are expressed as means ± SEM. Statistical analysis was accomplished by paired *t*-test, one-way ANOVA; followed by Tukey’s test, two-way ANOVA; followed by Tukey’s multiple comparisons test and two-way ANOVA; followed by Bonferroni’s multiple comparisons test. All statistical analyses were performed using Prism 9 (GraphPad Software, San Diego, CA, USA). *p* < 0.05 was considered significant.

## 3. Results

### 3.1. Effects of d-Allulose on Ghrelin-Induced [Ca^2+^]_i_ Increases in ARC Neurons

**Figure 1:**
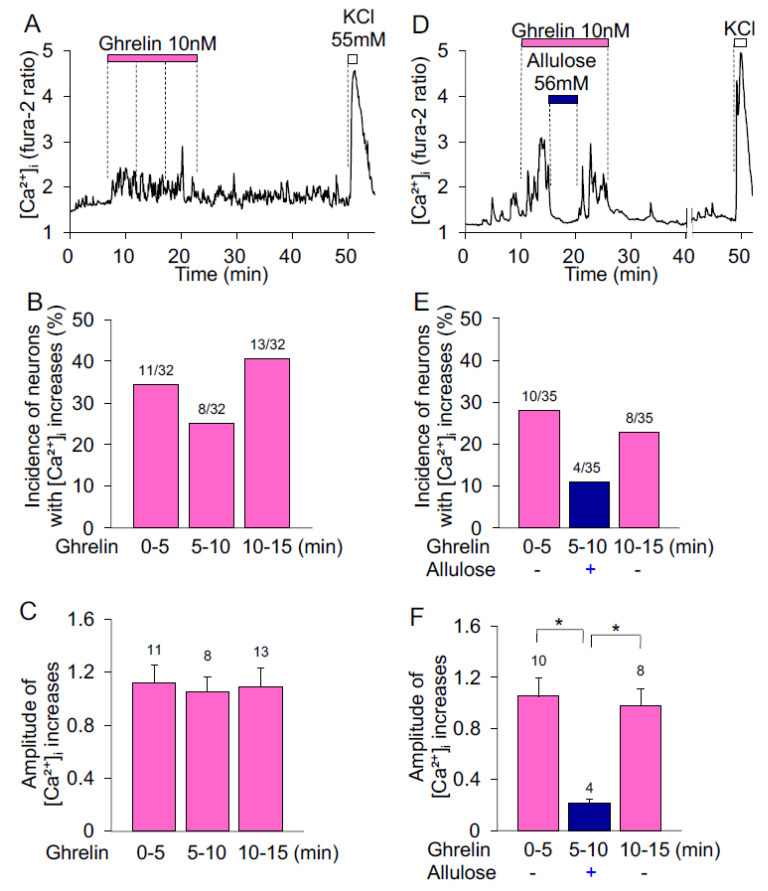
Effects of d-allulose on ghrelin-induced [Ca^2+^]_i_ increases in ARC neurons. [Ca^2+^]_i_ was measured in single neurons isolated from the hypothalamic ARC. Single neurons were superfused with HKRB containing 2 mM glucose. (**A**) Ghrelin (10 nM) was given for continuous 15 min. [Ca^2+^]_i_ started to increase during the first 0–5 min and continued to be increased during the middle 5–10 min and last 10–15 min periods of 15 min exposure to ghrelin. This neuron subsequently responded to KCl with an increase in [Ca^2+^]_i_. (**B**) Incidence of neurons with [Ca^2+^]_i_ increases during the first, middle and last periods of 15 min ghrelin exposure. The numbers above each bar indicate the number of neurons that showed [Ca^2+^]_i_ increases in response to ghrelin over that examined. (**C**) Amplitude of [Ca^2+^]_i_ increases during the first, middle and last periods of 15 min ghrelin exposure. Data are presented as mean ± SEM. No significant difference between groups by one-way ANOVA followed by Tukey’s test. (**D**) d-allulose was simultaneously administered in the middle period of 15 min ghrelin exposure. The ghrelin-induced [Ca^2+^]_i_ increase was inhibited by simultaneous administration of d-allulose in the middle period, and subsequently restored after washing d-allulose in the last period. This neuron responded to KCl with an increase in [Ca^2+^]_i_. (**E**) Incidence of the neurons with [Ca^2+^]_i_ increases in response to ghrelin in the absence (first and last periods) and presence (middle period) of d-allulose. The numbers above each bar indicate the number of neurons that showed [Ca^2+^]_i_ increases in response to ghrelin over that examined. (**F**) Amplitude of [Ca^2+^]_i_ increases in response to ghrelin in the absence (first and last periods) and presence (middle period) of d-allulose. All data are presented as mean ± SEM. * *p* < 0.05 by two-way ANOVA followed by Tukey’s multiple comparisons test.

Single neurons were isolated from ARC of the hypothalamus and [Ca^2+^]_i_ was measured under superfusion with HKRB containing 2 mM glucose. In control experiments, ghrelin was administered continuously for 15 min at 10 nM, the submaximal concentration in activating ARC NPY neurons [31]. In a neuron depicted in Figure 1A, [Ca^2+^]_i_ started to increase during the first 0–5 min period and continued to be elevated during the middle 5–10 min and the last 10–15 min periods of 15 min ghrelin administration. Ghrelin increased [Ca^2+^]_i_ in 11 of 32 (34%) neurons during the first period, in 8 of 32 (25%) neurons during the middle period, and in 13 of 32 (40%) neurons during the last period (Figure 1B). Amplitudes of [Ca^2+^]_i_ increases during the first, middle and last periods of 15 min ghrelin administration were not different from each other (Figure 1C).

d-allulose was simultaneously added in the middle period of ghrelin administration. The ghrelin-induced increases in [Ca^2+^]_i_ were depressed by d-allulose (56 mM) during the middle period and restored after washing d-allulose during the last period (Figure 1D). Out of 35 neurons, 10 neurons (28%) responded to ghrelin during the first period, 4 of 35 (11%) responded to ghrelin during the middle period under d-allulose treatment, and 8 of 35 (22%) responded to ghrelin during the last period after washing d-allulose (Figure 1E). The significant reduction in average amplitude of [Ca^2+^]_i_ increases was detected during treatment with d-allulose than before and after treatment (Figure 1F). These results indicate that d-allulose inhibits ghrelin-induced [Ca^2+^]_i_ increases in ARC neurons.

### 3.2. d-Allulose Effect on Low-Glucose-Induced [Ca^2+^]_i_ Increases in ARC Neurons

**Figure 2:**
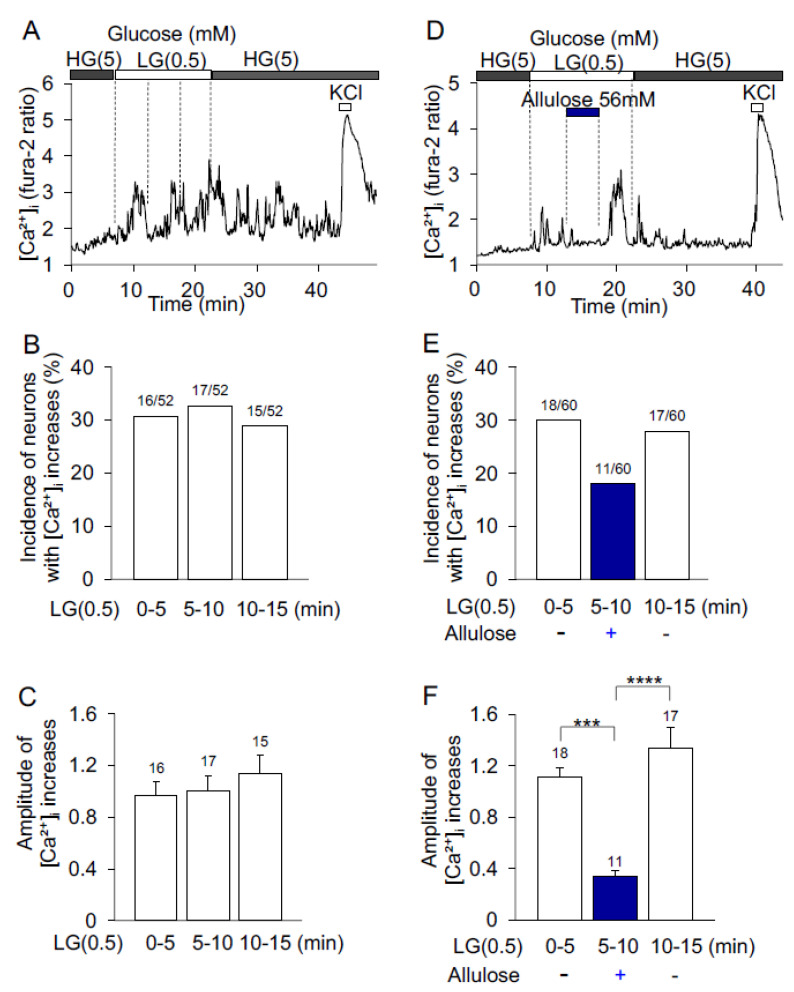
Effects of d-allulose on low glucose-induced [Ca^2+^]_i_ increases in ARC neurons. Single neurons were superfused with HKRB containing 5 mM glucose. (**A**) The superfusate HKRB containing high glucose (HG, 5 mM) was shifted to that containing low glucose (LG, 0.5 mM) for 15 min. [Ca^2+^]_i_ started to increase during the first 0–5 min and continued to be increased during the middle 5–10 min and last 10–15 min periods of 15 min LG exposure. This neuron subsequently responded to KCl with an increase in [Ca^2+^]_i._ (**B**) Incidence of ARC neurons with [Ca^2+^]_i_ increases during the first, middle and last periods of 15 min LG exposure. The numbers above each bar indicate the number of neurons that showed [Ca^2+^]_i_ increases in response to LG over that examined. (**C**) Amplitude of LG-induced [Ca^2+^]_i_ increases during the first, middle and last periods of 15 min LG exposure. Data are presented as mean ± SEM. No difference between groups by one-way ANOVA followed by Tukey’s test. (**D**) d-allulose was administered in the middle period of 15 min LG exposure. LG-induced [Ca^2+^]_i_ increase was inhibited by d-allulose administration in the middle period, and restored after washing d-allulose in the last period. This neuron subsequently responded to KCl with an increase in [Ca^2+^]_i._ (**E**) Incidence of the neurons with [Ca^2+^]_i_ increases in response to LG in the absence (first and last periods) and presence (middle period) of d-allulose. The numbers above each bar indicate the number of neurons that showed [Ca^2+^]_i_ increases over that examined. (**F**) Amplitude of [Ca^2+^]_i_ increases in response to LG in the absence (first and last periods) and presence (middle period) of d-allulose. All data are presented as mean ± SEM. *** *p* < 0.001, **** *p* < 0.0001 by two-way ANOVA followed by Tukey’s multiple comparisons test.

In control experiments, the superfusate HKRB containing high glucose (HG, 5 mM) was shifted to that containing low glucose (LG, 0.5 mM) for 15 min. In a neuron depicted in Figure 2A, [Ca^2+^]_i_ increase started during the initial 0~5 min period and continued during the middle 5~10 and last 10~15 min periods of 15 min exposure to LG. LG increased [Ca^2+^]_i_ in 16 of 52 (30%), 17 of 52 (32%), and 15 of 52 (28%) neurons during the first, middle and last periods, respectively (Figure 2B). Amplitude of LG-induced [Ca^2+^]_i_ increases during the first, middle and last periods were not different from each other (Figure 2C).

d-allulose was administered during the middle period of 15 min LG exposure. The LG-induced increases in [Ca^2+^]_i_ were suppressed by administration of d-allulose (56 mM) during the middle period, and restored after washing d-allulose during the last period (Figure 2D). Among 60 neurons, 18 neurons (30%) responded to LG during the first period, 11 (18.3%) responded to LG under d-allulose treatment during the middle period, and 17 (28.3%) responded to LG after washing d-allulose during the last period (Figure 2E). During the treatment with d-allulose, the average amplitude of [Ca^2+^]_i_ increases was significantly smaller than before and after treatment (Figure 2F). These data indicate that d-allulose inhibits LG-induced [Ca^2+^]_i_ increases in glucose-sensitive ARC neurons.

### 3.3. Effects of d-Allulose on Spontaneous [Ca^2+^]_i_ Increases in NPY Neurons

**Figure 3:**
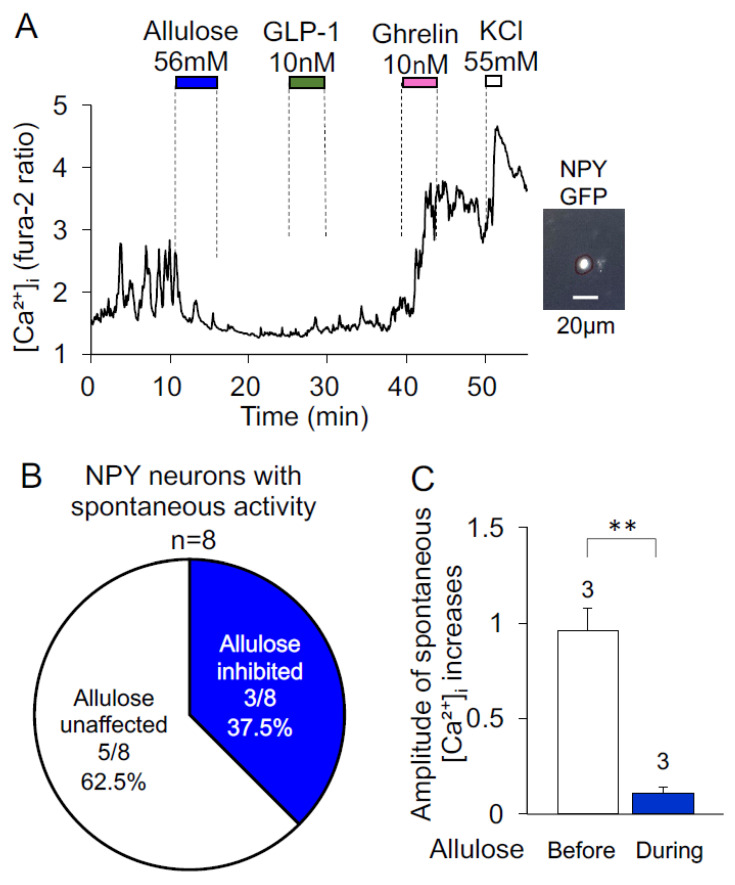
d-allulose effect on spontaneous [Ca^2+^]_i_ increases in NPY neurons isolated from NPY-GFP mice. [Ca^2+^]_i_ was measured under superfusion with HKRB containing 2 mM glucose. (**A**) Spontaneous oscillatory increases in [Ca^2+^]_i_ were inhibited by d-allulose (56 mM) in an NPY neuron (Left) identified by GFP fluorescence (Right). This neuron subsequently responded to KCl with an increases in [Ca^2+^]_i_. (**B**) Incidence of inhibition by d-allulose of NPY neurons with spontaneous [Ca^2+^]_i_ increases. The numbers in a circle graph indicate the number of NPY neurons that were inhibited by d-allulose over that exhibiting spontaneous [Ca^2+^]_i_ increases. (**C**) Amplitude of spontaneous [Ca^2+^]_i_ increases before and during treatment with d-allulose in d-allulose-inhibited NPY neurons. All data are presented as mean ± SEM. ** *p* < 0.01 by paired *t*-test.

As depicted in Figure 3A, d-allulose (56 mM) administration inhibited oscillatory [Ca^2+^]_i_ increases that occurred spontaneously in an NPY neuron, as identified by NPY-GFP fluorescence. The neuron afterward responded to ghrelin (10 nM) and KCl with [Ca^2+^]_i_ increases, but did not respond to GLP-1. d-allulose inhibited spontaneous [Ca^2+^]_i_ increases in three of eight (37.5%) NPY neurons (Figure 3B). These three neurons showed significantly smaller average amplitude of [Ca^2+^]_i_ increases during treatment with d-allulose than before treatment (Figure 3C). These results show that d-allulose inhibits the [Ca^2+^]_i_ activity in a part of ARC NPY neurons with spontaneous oscillatory [Ca^2+^]_i_ increases.

### 3.4. Effects of Central Injection of d-Allulose on Feeding

**Figure 4:**
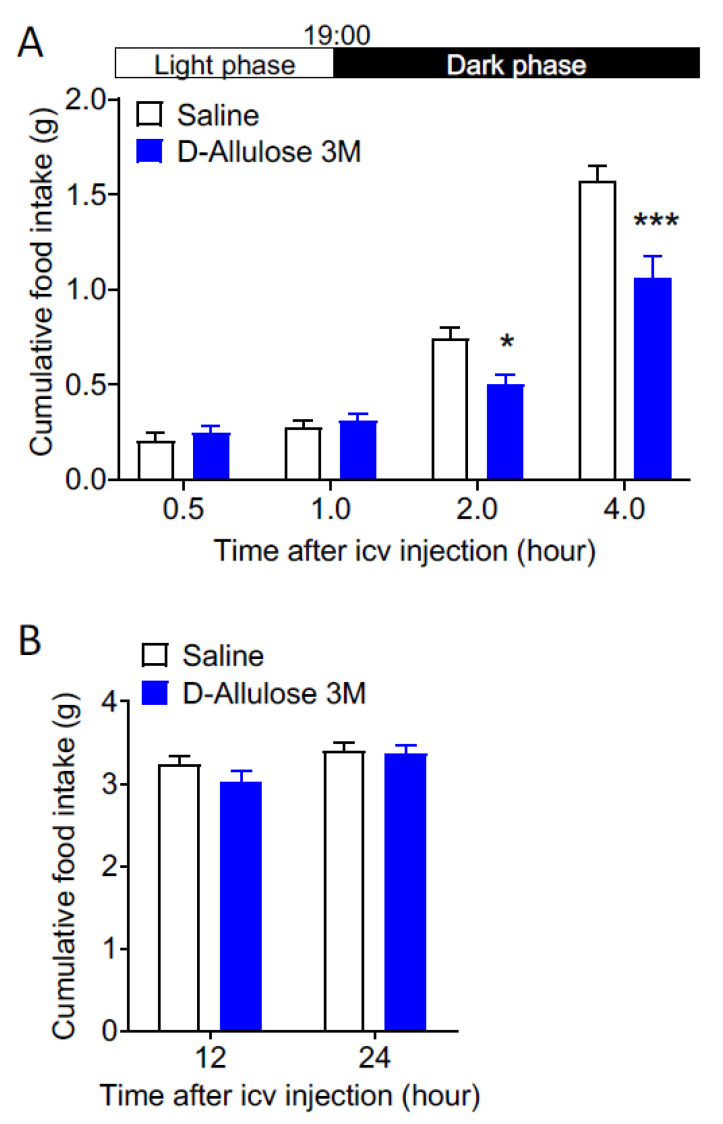
Icv injection of d-allulose inhibits feeding in the early dark phase in mice. Saline (0.9% NaCl, 3 μL; control) or d-allulose (3 M, 3 μL) was icv-injected at 18:00 in mice fasted 11 h. (**A**) d-allulose had no effect on cumulative food intake until 19:00 in light phase, and significantly decreased cumulative food intake at 20:00 and 22:00 in the early dark phase at 2 and 4 h after icv injection. Data are presented as mean ± SEM. * *p* < 0.05 and *** *p* < 0.001 by two-way ANOVA followed by Bonferroni’s multiple comparisons test, *n* = 7–8. (**B**) d-allulose altered cumulative food intake neither at the next day 6.00 (12 h after icv injection) in the late dark phase nor at 18.00 (24 h after icv injection) in light phase. Data are presented as mean ± SEM. No difference was observed between groups by two-way ANOVA followed by Bonferroni’s multiple comparisons test, *n* = 7–8.

d-allulose (3 M, 3 μL) or saline (0.9% NaCl, 3 μL) was icv-injected at 18:00 in mice fasted for 11 h. d-allulose had no effect on cumulative food intake until 19:00 in the light phase. In contrast, d-allulose significantly suppressed cumulative food intake at 20:00 and 22:00 in the early dark phase at 2 and 4 h after icv injection (Figure 4A), without affecting cumulative food intake at 12 and 24 h after icv injection (Figure 4B). These results indicate that centrally administered d-allulose significantly inhibits food intake in the early dark phase in mice.

## 4. Discussion

The present study showed that d-allulose via direct action decreases [Ca^2+^]_i_ in ghrelin-responsive neurons, glucose-sensitive neurons and NPY neurons in the ARC. These neuron types, partly overlapping with each other, play a pivotal role in triggering hunger and promoting feeding behavior [20,21,22,23,24,25,26,27,28]. [Ca^2+^]_i_ regulates neuronal activities, including synaptic activity, neurotransmitter release and gene expression [34,35,36]. Therefore, the results of the present study demonstrate that d-allulose inhibits the orexigenic neurons in the ARC that are implicated in hunger. Moreover, icv-injected d-allulose decreased food intake at 20:00 and 22:00, the beginning of the dark phase when feeding behavior is promoted by hunger. The inhibition of ARC ghrelin-responsive, glucose-sensitive and NPY neurons by d-allulose may serve as a mechanism by which centrally injected d-allulose suppresses feeding in the early dark phase. The particular cellular and molecular mechanisms underlying the d-allulose action to inhibit these neurons remain unknown. However, ghrelin and LG increase [Ca^2+^]_i_ in ARC neurons via the mechanisms involving AMPK, protein kinase A, N-type Ca^2+^ channel and Na^+^-K^+^ ATPase [31,37,38]. d-allulose tastes sweet, and sweet taste receptors are localized in several organs including the hypothalamus [39]. Hence, these molecules are the potential targets for the inhibitory action of d-allulose in ARC neurons. Regarding the sweet taste receptors, a sweet taste receptor antagonist, lactisole, was shown not to significantly inhibit d-allulose action to stimulate release of GLP-1, CCK and PYY [40,41], suggesting that these receptors are not involved in the d-allulose-induced release of gut hormones. However, it does not exclude the possible involvement of sweet taste receptors in the d -allulose action on the ARC orexigenic neurons.

d-allulose, orally taken, is mostly absorbed from the gut into the blood circulation [9]. Whether d-allulose in the blood circulation could enter the brain by passing through the blood–brain barrier (BBB) remains unsolved. An autoradiographic study using ^14^C-labeled d-allulose did not support a substantial passage of d-allulose into the brain through the BBB [42]. Nevertheless, it was reported recently that some neurons in the ventromedial hypothalamic ARC are not secluded by the BBB and are sensitive to peripheral signals that are transported through the tanycyte pathway [43]. The tanycytes are able to transport physiologic and pharmacologic substances in the circulation to the third ventricle and the ARC. The substances transported by this pathway include leptin [44], thyroid hormone [45,46], insulin [47] and the GLP-1 receptor agonist [48], and the underlying mechanism involves adaptor proteins such as receptors expressed in tanycytes. The GLUT5, the feasible transporter for d-allulose, is expressed in the brain areas where tanycytes are located [49]. A recent study using electron microscopy detected GLUT5 immunoreactive deposition on the tanycyte processes [50]. Based on these findings, we hypothesize that d-allulose can be transported to the ARC through the tanycyte pathway to exert direct central action.

In this study, d-allulose decreased [Ca^2+^]_i_ in 28% of ghrelin-responsive and 30% of glucose-sensitive neurons, and reduced spontaneous [Ca^2+^]_i_ increases in 37.5% of NPY neurons. These results show that d-allulose inhibits significant proportion of the ARC neurons implicated in hunger. The direct inhibition of these hunger-associated neurons by d-allulose may underlie the action of icv-injected d-allulose to reduce food intake in the early dark phase when hunger is promoted.

The ARC NPY neurons and POMC neurons are the principal orexigenic and anorexigenic neurons, respectively, and counteract each other in regulating feeding and energy expenditure. Hence, the substances that reciprocally regulate these two neurons are considered efficient in normalizing energy balance. However, such substances are rare, except for a few hormones including leptin and insulin that activate POMC neurons and inhibit NPY neurons in the ARC [51,52,53]. Our present results, together with previous report [18], reveal that d-allulose inhibits NPY neurons and activates POMC neurons in the ARC, and thereby place d-allulose as a dual and reciprocal regulator of the principal orexigenic and anorexigenic neurons. This property represents a promising potential of d-allulose to attenuate hyperphagia owing to both excessive appetite and reduced satiety, and thereby to efficiently remedy obesity and diabetes.

## Data Availability

All data are available in this paper.
